# The role of cytology in patients undergoing pressurized intraperitoneal aerosol chemotherapy (PIPAC) treatment for peritoneal carcinomatosis

**DOI:** 10.1515/pp-2022-0197

**Published:** 2023-05-08

**Authors:** Mélina Deban, Julien Châtelain, François Fasquelle, Daniel Clerc, Laura Toussaint, Martin Hübner, Hugo Teixeira Farinha

**Affiliations:** Department of Visceral Surgery, Faculty of Biology and Medicine UNIL, Lausanne University Hospital (CHUV), Lausanne, Switzerland; Section of Surgical Oncology, Department of Surgery, University of Calgary, Calgary, Canada; Faculty of Biology and Medicine UNIL, Institute of pathology, Lausanne University Hospital (CHUV), Lausanne, Switzerland

**Keywords:** cytology, peritoneal cancer, peritoneal surface malignancies, pressurized intraperitoneal aerosol chemotherapy (PIPAC)

## Abstract

**Objectives:**

Cytology of ascites or peritoneal washing is a routine part of staging of peritoneal metastases (PM). We aim to determine value of cytology in patients undergoing pressurized intraperitoneal aerosol chemotherapy (PIPAC).

**Methods:**

Single-center retrospective cohort study included consecutive patients having PIPAC for PM of different primary between January 2015 and January 2020.

**Results:**

A total of 75 patients (median 63 years (IQR 51–70), 67 % female) underwent a total of 144 PIPAC. At PIPAC 1 59 % patients had positive and 41 % patients had negative cytology. Patients with negative and positive cytology only differed in terms of symptoms of ascites (16% vs. 39 % respectively, p=0.04), median ascites volume (100 vs. 0 mL, p=0.01) and median PCI (9 vs. 19, p<0.01). Among 20 patients who completed 3 PIPACs (per protocol), cytology changed in one from positive to negative, and in two from negative to positive. Median overall survival was 30.9 months in the per protocol group and 12.9 months in patients having <3 PIPACs (=0.519).

**Conclusions:**

Positive cytology under PIPAC treatment is more frequently encountered in patients with higher PCI and symptomatic ascites. Cytoversion was rarely observed and cytology status had no impact on treatment decisions in this cohort.

## Introduction

Treatment of peritoneal surface malignancies (PSM) is a challenging frontier for a variety of malignancies, with few therapeutic options and a limited prognosis [1]. Pressurized intraperitoneal aerosol chemotherapy (PIPAC) is a novel minimally invasive treatment for patients with advanced peritoneal metastases, which allows repetitive administration of cytostatics within the abdominal cavity. Preclinical data suggest improved distribution and tissue penetration of IP chemotherapy through pressurized aerosolization compared with catheter-based administration or lavage [2, 3]. Recent systematic reviews confirmed feasibility, safety, and excellent tolerance of PIPAC overall in patients with PSM [2, 3]. Regarding clinical outcomes, objective clinical response was reported after PIPAC in 62–88 % of patients with ovarian cancer (median survival of 11–14 months), 50–91 % for gastric cancer (median survival of 8–15 months), 71–86 % for colorectal cancer (median survival of 16 months) [2].

Based on expert consensus, the current protocol consists of three PIPAC procedures planned every 4–6 weeks [2, 3]. Repeated procedures allow direct evaluation of treatment response using the peritoneal cancer index (PCI). With biopsies performed at each procedure, the pathologist evaluates the peritoneal regression grading score (PRGS) [4]. Cytology obtained by peritoneal lavage or ascites sampling is also part of routine practice during PIPAC procedures [2]. By contrast to PCI and PRGS, the role of cytology in patient care management remains unclear [5].

The aim of this study was therefore to determine the value of cytology in the algorithm of PIPAC treatment.

## Materials and methods

A retrospective cohort study was performed in consecutive patients having undergone PIPAC treatment at Lausanne University Hospital (CHUV, Switzerland) between January 2015 and January 2020. Included patients had peritoneal cancer (PC) from various origins, namely colorectal, gastric, ovarian, hepatobiliary and mesothelioma. Indications for PIPAC treatment were discussed during in the institution’s multidisciplinary tumor board. The study was approved by the Institutional Review Board CER-VD 2019-00747.

Patients were separated in two groups: positive and negative cytology at PIPAC 1. Evolution of cytology was assessed during the 3 PIPAC procedures.

Data including demographics, previous systemic IV chemotherapy, symptoms before PIPAC, surgical details and postoperative complications were compared between cytology-positive and – negative patients. Evolution of cytology from PIPAC 1 to PIPAC 3 was collected.

### Data acquisition

Demographics, surgical and oncological data were retrieved from prospectively maintained institutional databases. The following variables were extracted: gender, age, primary tumor origin, ASA score, ECOG Performance Status Scale [6], previous number of cycles and lines of systemic chemotherapy, presence of symptoms before PIPAC (abdominal pain, ascites, obstructive symptoms, nausea), PCI (Peritoneal Cancer Index) [7], Peritoneal Regression Grading Score (PRGS) [4], postoperative complications described using the Clavien classification [8], and median overall survival (OS).

### PIPAC procedure and safety considerations

All procedures were performed in a standardized manner according to the PIPAC certification course and in line with recent international expert consensus [9, 10]. In the absence of ascites, approximately 100 cc of saline were instilled in the right and left hypochondriums, in addition to 100 cc in the pelvis. Saline was suctioned and sent for cytology. Oxaliplatin was applied at a dose of 92 mg/m^2^ for carcinomatosis of colorectal origin. Cisplatin (7.5 mg/m^2^) in combination with doxorubicin (1.5 mg/m^2^) was initially used for ovarian, gastric, and other malignancies. Starting from 2019, a dose adaptation (10.5 mg/m^2^ and 2.1 mg/m^2^) occurred in line with recommendations based on current literature [11].

### Processing of cytology in pathology

Serous fluid was obtained by peritoneal lavage (as described above) or by sampling of ascites when present immediately before any tumoral manipulation and biopsies.

Immediately after the abdominal cavity had been opened, and before any manipulation of the tumour, serous fluid was obtained by peritoneal washing or by sampling of ascites when present. The cytological examination was based on three smears stained according to Papanicolaou, and one using May–Grunwald Giemsa (MGG) stain. The cytological examination result was considered positive if malignant tumour cells were detected.

### Statistical analysis

Continuous variables are presented as mean with standard deviation (SD) or median with interquartile range (IQR) according to their distribution. Categorical variables are reported as frequencies (%) and compared with chi-square test. Student’s *t*-test or Mann–Whitney test were used to compare continuous variables. All statistical tests were two-sided and a level of 0.05 was used to indicate statistical significance. Statistical analyses were performed with GraphPad Prism 8 (GraphPad Software, Inc., La Jolla, CA, USA).

## Results

In the study period, 75 patients underwent a total of 144 PIPAC procedures. At PIPAC 1, 44 patients (59 %) had positive and 31 patients (41 %) had negative cytology, respectively. Approximately 40 % of PIPACs was performed in patients with ovarian malignancies, the second most common site of origin being colorectal. Between 35% to 41 % of patients had received 3 or more lines of systemic chemotherapy and over 80 % had received 12 or more cycles. There was no imbalance in terms of demographical or clinical characteristics between patients with negative cytology and those with positive cytology, with the exception of symptoms prior to PIPAC. Significantly more patients with positive cytology had symptoms of ascites (5 (16 %) vs. 17 (39 %), p=0.04). Similarly, 6 patients (14 %) with positive cytology had undergone drainage of ascites within 4 weeks prior to PIPAC vs. none in the negative cytology group (Table 1).

**Table 1:** Demographics of patients with negative and positive cytology.

	Negative cytology at PIPAC#1 n=31 patients	Positive cytology at PIPAC#1 n=44 patients	p-Value
Demographics			
Median age (IQR)	63 (48–71)	62 (54–69)	0.99
Gender male, n (%)	11 (35 %)	14 (32 %)	0.71
ASA III-IV, n (%)	6 (19 %)	16 (36 %)	0.19
ECOG 0-I, n (%)	21 (68 %)	35 (80 %)	0.28
Disease origin			0.52
Ovarian, n (%)	13 (42 %)	17 (39 %)	
Colorectal, n (%)	9 (29 %)	15 (34 %)	
Gastric, n (%)	1 (3 %)	1 (2 %)	
Hepatobiliary, n (%)	2 (6 %)	5 (11 %)	
Mesothelioma, n (%)	6 (20 %)	6 (14 %)	
Previous systemic chemotherapy			
≥3 lines in total	11 (35 %)	18 (41 %)	0.63
≥12 cycles in total	29 (93 %)	35 (80 %)	0.45
Symptoms before PIPAC procedures			
Abdominal pain, n (%)	3 (10 %)	10 (23 %)	0.22
Ascites, n (%)	5 (16 %)	17 (39 %)	**0.04**
Obstructive symptoms (ileus), n (%)	4 (13 %)	2 (5 %)	0.22
Ascites drainage within last 4 weeks before PIPAC, n (%)	0	6 (14 %)	

Median (IQR, interquartile rang or range), mean (SD, standard deviation) or number (%) as appropriate. Statistical significance (p<0 05) is highlighted in bold. ASA, American Association of Anesthesiologists physical status classification system. ECOG Performance Status Scale.

With regards to surgical details, there was no difference in number of PIPAC treatments between patients with negative and positive cytology. Most patients had 2 PIPAC procedures, and 20 patients (27 %) completed 3 PIPACs (per protocol). Median ascites volume was significantly higher for patients with positive cytology (0 vs. 100 mL, p=0.01). Median PCI was also greater in patients who had positive cytology (19 vs. 9, p<0.01). PRGS and intraoperative complications did not differ between the two groups. Ascites was negative for malignancy in 9 patients while peritoneal lavage detected malignancy in 17 patients (Table 2).

**Table 2:** Surgical treatment of patients with negative and positive cytology.

	Negative cytology at PIPAC#1 n=31 patients	Positive cytology at PIPAC#1 n=44 patients	p-Value
Surgical details			
Number PIPAC per patients, n (%)			0.23
1–2 PIPAC	25 (80 %)	30 (68 %)	
3 PIPAC	4 (13 %)	11 (25 %)	
>3 PIPAC	2 (6 %)	3 (7 %)	
per protocol (≥3 PIPAC)	6 (19 %)	14 (32 %)	0.23
Median ascites volume during PIPAC, mL (IQR)	0 (0–100)	100 (0–1,075)	**0.01**
Median PCI (IQR)	9 (3–18)	19 (11–27)	**<0.01**
Mean of PGRS, median (IQR)	2 (1–2)	2 (2–3)	0.10
Intraoperative complications, n (%)	2 (6 %)	2 (5 %)	0.44
Cytology origin			0.01
Ascites, n (%)	9 (29 %)	27 (62 %)	
Peritoneal lavage, n (%)	22 (71 %)	17 (38 %)	

Median (IQR, interquartile rang or range), mean (SD, standard deviation) or number (%) as appropriate. Statistical significance (p<0 05) is highlighted in bold. ASA, American Association of Anesthesiologists physical status classification system; ECOG Performance Status Scale; PCI, Peritoneal Cancer Index; PRGS, Peritoneal Regression Grading Score.

### Changes in cytology

**Figure 1:**
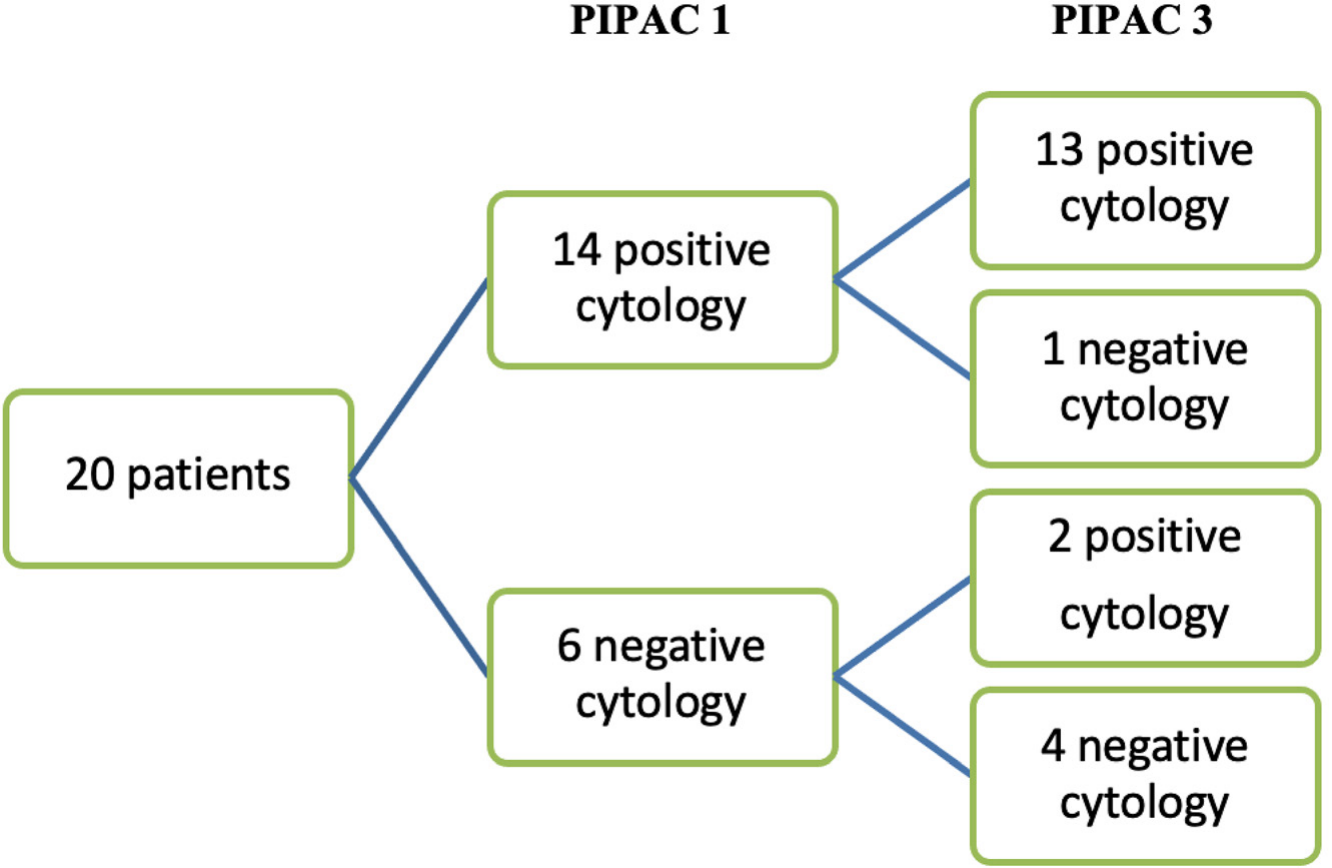
Flowchart of changes in cytology between PIPAC 1 and 3 (per protocol patients).

In patients who completed the protocol consisting of 3 PIPAC treatments, one patient with an initially positive cytology was converted to negative cytology. In 6 patients with negative cytology, two became positive by the time PIPAC 3 was performed (Figure 1).

**Figure 2:**
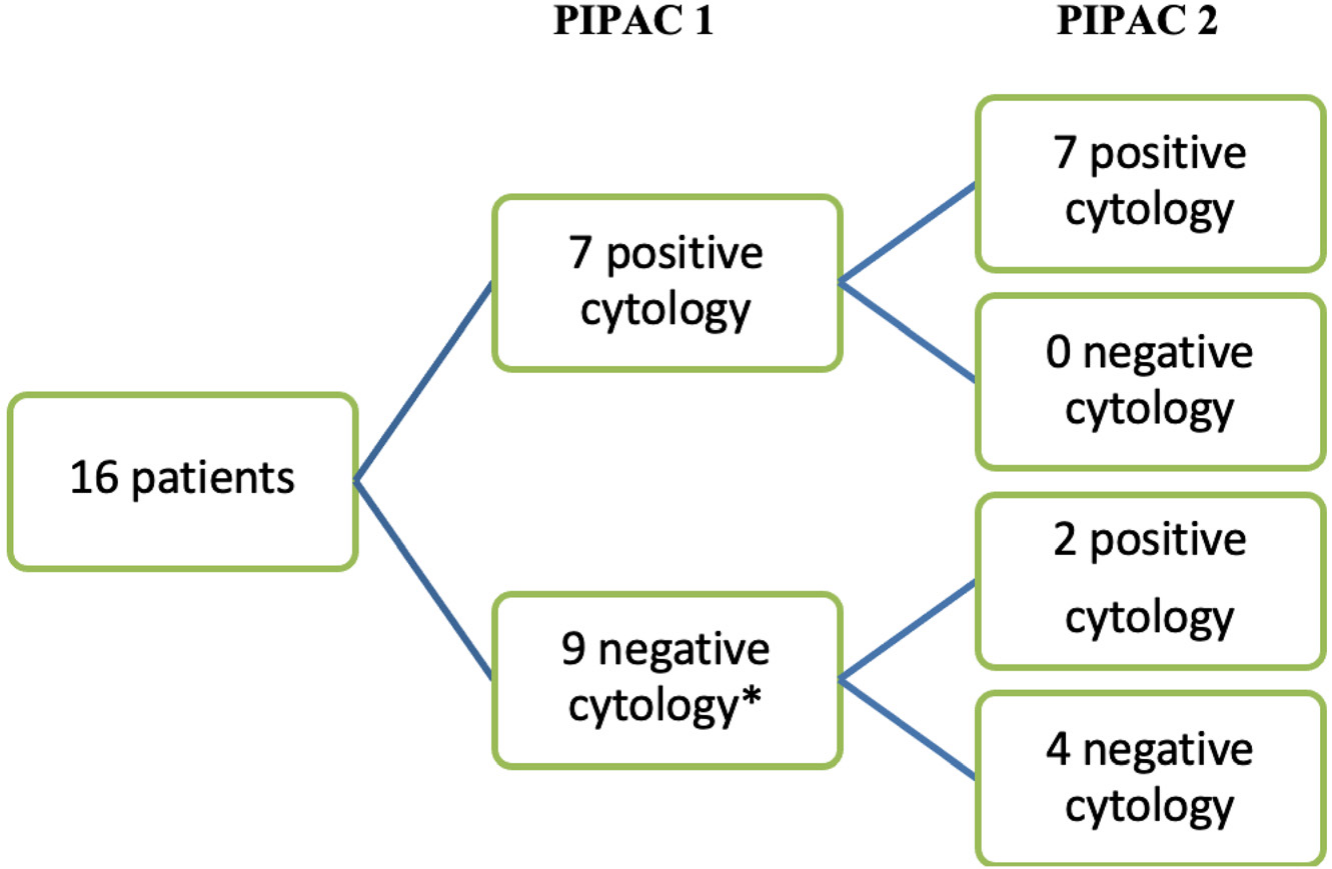
Flowchart of changes in cytology between PIPAC 1 and 2 (≠ per protocol patients). * 3 patients with unknown cytology at PIPAC 2.

Out of 16 patients who had 2 PIPAC treatments, 2 with an initially negative cytology became positive at PIPAC 2. None of the patients with a positive cytology was converted to negative at PIPAC 2 (Figure 2).

### Changes in cytology: details of clinical cases

For two patients with baseline negative cytology who completed the protocol of 3 PIPAC treatments conversion was observed to positive cytology. The first patient was female, with PC of ovarian origin and an initial PCI of 18. No ascites was found during all the three procedures and cytology was obtained by peritoneal lavage. Cytology became positive at PIPAC 2. PRGS at PIPAC 3 was 1.5. After completion of the 3 procedures, the patient experienced peritoneal progression with bowel obstruction. She died 10 months after the last PIPAC procedure.

The second patient was female, with PC of colorectal origin and an initial PCI of 25. Cytology was obtained by ascites suctioning (approximately 500 mL during each PIPAC). Cytology became positive at PIPAC 2. PRGS at PIPAC 3 was 2.5. 13 months after completion of the third procedure, the patient had peritoneal progression. After discussion at the multidisciplinary tumor board, the decision was made to pursue treatment only with palliative chemotherapy. She was lost to follow-up 15 months after the last PIPAC. Cytology was not taken into account as an argument to continue or discontinue PIPAC for these two patients.

One patient who completed the protocol of 3 PIPAC treatments had initially positive cytology with conversion to negative cytology. She was female, with PC of ovarian origin. The initial PCI was 24. Ascites was found during all three procedures (between 2,500 and 3,000 mL) and was the source of cytology analysis. Cytology became negative at PIPAC 3. PRGS at PIPAC 3 was not assessed. After completion of the 3 procedures, she continued follow-up with the oncological team. The patient had a radiological peritoneal progression 19 months after the last PIPAC. Decision was made to treat with systemic chemoterapy.

None of the 3 patients with changes in cytology who completed all 3 procedures had bidirectional treatment. Regarding the two patients with an initially negative cytology who became positive at PIPAC 2, the reasons to stop before PIPAC 3 were the patient’s wish to stop PIPAC and extra-peritoneal progression. The two patients had PC from ovarian origin.

### Median overall survival

**Figure 3:**
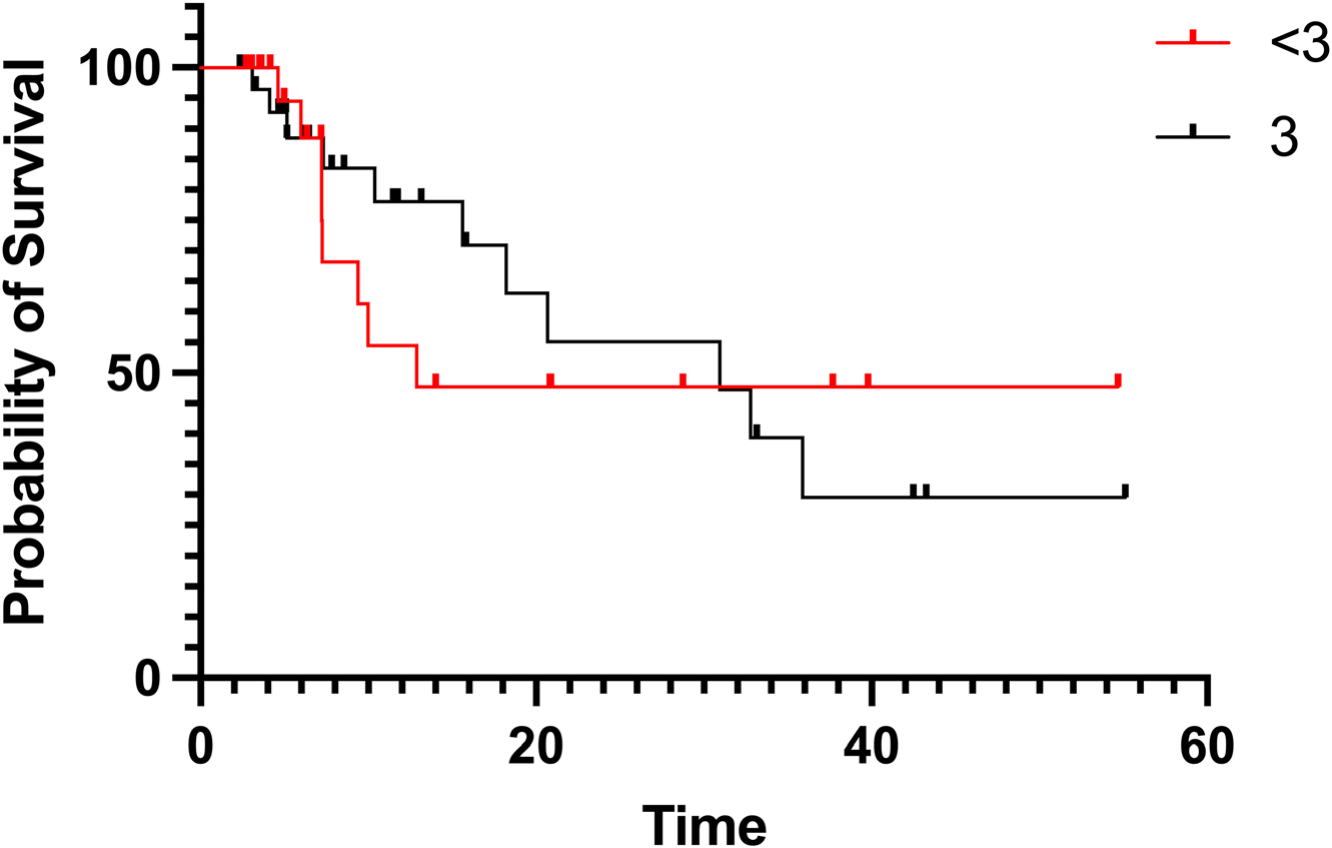
Median overall survival of per protocol patients vs. patients having completed less than 3 PIPACs.

Median OS was compared between the per protocol group and the group having completed less than 3 PIPACs. Though median OS was numerically superior for the per protocol group (30.9 vs. 12.9 months), it did not achieve statistical significance (p=0.519) (Figure 3).

## Discussion

In this study, positive cytology was frequently observed in PIPAC patients with high PCI and symptomatic ascites. However, cytology status changed rarely under treatment and did not impact treatment decisions for patients included in this study.

Positive cytology was most often noted in patients with symptoms of ascites, occasionally prompting drainage within 4 weeks prior to the PIPAC procedure. Moreover, a higher PCI was noted in these patients. This result likely reflects the reality of more advanced disease and malignant ascites, to which analysis of cytology arguably adds little clinical value [12]. In other words, in patients who have extensive disease, of which malignant ascites is a symptom, cytology might be irrelevant.

On the other hand, 17 patients had a positive finding of malignancy after peritoneal lavage despite the absence of ascites. The presence of intraperitoneal free cancer cells has been shown to portend peritoneal recurrence and worse prognosis [13, 14] in gastric cancer. Similarly, in colorectal cancer, it represents a major prognostic factor linked to poor survival [15, 16]. This is perhaps more relevant in the context of a tumor with limited locoregional extent for which standard curative management in planned; however, with gross peritoneal disease for which treatment is already underway, management is likely not affected by the presence of free intraperitoneal cells.

The role of cytology in PIPAC has been examined in a paper published by Benzerdjeb and colleagues in 2020 [5]. They defined a combined progression index, made from the peritoneal regression grading score (PRGS) and peritoneal cytology. This index, when using highest PRGS (as opposed to mean), was independently associated with overall survival and progression-free survival. It is unclear to which extent the cytology component of the score contributed to this finding.

Graversen and colleagues [17] evaluated cytology samples from 35 patients with peritoneal carcinomatosis of various origins. They found that conventional cytology has low sensitivity in patients with peritoneal carcinomatosis. The sensitivity can be improved with carcinoembryonic antigen and epithelial cell adhesion molecule mRNA assays. However, they did not find any significant relation between cytology findings and response to PIPAC treatment.

The current study does not diminish the role of peritoneal lavage and cytology as a staging procedure. As mentioned earlier, peritoneal lavage can detect free intraperitoneal cells, which are an established indicator of worse prognosis. What is less certain is the significance of such a result in the context of known peritoneal metastasis with planned peritoneal treatment. Moreover, no consensus or guideline exists on the technique of cytology, for instance how many milliliters should be instilled or how long it should stay in the abdomen before suctioning. The clinical scenarios described the fact that the number of PIPAC treatments was similar regardless of cytology and the minimal rate of conversion from positive to negative suggests that the value of cytology assessment in this precise background is little to none.

The scenarios where cytology could potentially change the treatment strategy in the context of PSM, are the following: (I) positive cytology in the absence of overt peritoneal metastases at surgical exploration in patients at high risk=microscopic peritoneal disease, (II) cytology status in patients with complete histopathological response after treatment=determinant for complete response yes/no, (III) conversion from cytology negative to positive under treatment=indicator for non-response and argument to switch therapy. None of these conditions was met in any patient of this cohort.

The main objective of this study was to assess the role of cytology in treatment decision in PIPAC. It was not designed to address treatment efficacy. Recent systematic reviews suggested objective treatment response in about 60 % of patients in mostly mixed cohorts of patients [2]. The international PIPAC cohort study analyzed very recently treatment response and survival under PIPAC treatment by disease entity [18, 19] reporting survival curves well beyond published survival by the standard treatment. Important ongoing studies are NCT04734691for colorectal, NCT04065139 for gastric, NCT04811703, NCT04000906 for ovarian, and NCT03875144 for mesothelioma.

In the literature, only about 50 % of patients benefit of the complete treatment of 3 PIPAC procedures [2]. The reasons for stopping are multifold as in our cohort. Recent published results suggested that the absence of ascites and prior bowel obstruction increases the chances of completing the three PIPAC procedures and best results seem to be achieved when PIPAC is combined with systemic chemotherapy [20]. Moreover, current accepted indications of PIPAC are mainly palliative, which raises the arduous question of which endpoints are most adequate to study treatment efficacy. It is undetermined if OS, recurrence-free survival, radiologic/PCI/pathologic changes, or patient-centered quality of life questionnaires is the best-suited endpoint for this purpose. Multiple excellent studies are underway to evaluate PIPAC treatment efficacy and will hopefully answer this question [3]. Within the framework of the international PIPAC cohort studies, current efforts are focused on the elaboration and validation of a predictive score for optimized patient selection for PIPAC treatment.

Limitations of this study include its relatively small and heterogeneous cohort population. Five different disease origins are included, and chemotherapy regimens vary widely. Focusing on one disease origin would have significantly restricted our population size, with loss of valuable information. Few patients completed the local protocol of 3 consecutive PIPACs, leaving a small sample to evaluate the conversion of cytology. This is a difficulty faced by many centers in an era where PIPAC is relatively new and under exploration. In addition, the monocentric nature of the cohort might render interpretation of results difficult in a wider context. However, it included patients for which all parameters were entered prospectively and systematically in a centralized database. As discussed before, no consensus or guideline exist on the technique of cytology. According to published literature [2, 17] and institutional standards, cytology smears are typically performed. Recently, cell blocks have been proposed as a new cytology technique. Cell blocks are prepared from residual tissue fluids and can be useful complements to smears for a more complete cytopathologic diagnosis, particularly in cases where tumors cannot be adequately categorized by smears alone [21, 22]. This technique could be used more frequently in the future, including for PIPAC patients for the detection of residual disease.

To our knowledge, this study is the first one to focus solely on the value of cytology as a factor in the management of patients who underwent one or several PIPAC procedures. Additional studies are necessary to definitively rule out the need to perform cytology for each PIPAC procedure.

## Conclusions

In conclusion, the present study demonstrates that cytology had little value in the context of PIPAC procedures, suggesting that foregoing peritoneal lavage would not alter clinical management. While there is a prognostic value to cytology in tumors with locoregional extent, in the context of established peritoneal disease under treatment, correlation with other validated tools such as the PGRS and PCI is advised. This would result in a simplified procedure, with gains in time, resources, and costs.
